# Defining the Boundaries of Normal Thrombin Generation: Investigations into Hemostasis

**DOI:** 10.1371/journal.pone.0030385

**Published:** 2012-02-02

**Authors:** Christopher M. Danforth, Thomas Orfeo, Stephen J. Everse, Kenneth G. Mann, Kathleen E. Brummel-Ziedins

**Affiliations:** 1 Department of Mathematics and Statistics, Center for Complex Systems, Vermont Advanced Computing Center, University of Vermont, Burlington, Vermont, United States of America; 2 Department of Biochemistry, College of Medicine, University of Vermont, Burlington, Vermont, United States of America; Institut National de la Santé et de la Recherche Médicale, France

## Abstract

In terms of its soluble precursors, the coagulation proteome varies quantitatively among apparently healthy individuals. The significance of this variability remains obscure, in part because it is the backdrop against which the hemostatic consequences of more dramatic composition differences are studied. In this study we have defined the consequences of normal range variation of components of the coagulation proteome by using a mechanism-based computational approach that translates coagulation factor concentration data into a representation of an individual's thrombin generation potential. A novel graphical method is used to integrate standard measures that characterize thrombin generation in both empirical and computational models (e.g max rate, max level, total thrombin, time to 2 nM thrombin (“clot time”)) to visualize how normal range variation in coagulation factors results in unique thrombin generation phenotypes. Unique ensembles of the 8 coagulation factors encompassing the limits of normal range variation were used as initial conditions for the computational modeling, each ensemble representing “an individual” in a theoretical healthy population. These “individuals” with unremarkable proteome composition was then compared to actual normal and “abnormal” individuals, *i.e.* factor ensembles measured in apparently healthy individuals, actual coagulopathic individuals or artificially constructed factor ensembles representing individuals with specific factor deficiencies. A sensitivity analysis was performed to rank either individual factors or all possible pairs of factors in terms of their contribution to the overall distribution of thrombin generation phenotypes. Key findings of these analyses include: normal range variation of coagulation factors yields thrombin generation phenotypes indistinguishable from individuals with some, but not all, coagulopathies examined; coordinate variation of certain pairs of factors within their normal ranges disproportionately results in extreme thrombin generation phenotypes, implying that measurement of a smaller set of factors may be sufficient to identify individuals with aberrant thrombin generation potential despite normal coagulation proteome composition.

## Introduction

The coagulation of blood is the initial phase of the biological repair process that responds to perforating trauma to the vasculature; its function is to stop blood loss from the circulatory system by establishing a temporary barrier between the intra- and extra-vascular compartments. The enzyme thrombin is a central product of the response to vascular injury, displaying procoagulant, anticoagulant, fibrinolytic and cellular effects; the magnitude and timing of its effects are critical to normal hemostasis [1].

Relatively unique levels of detail are available for this biological network concerning its cellular and protein components, connections between these components, and the dynamics characterizing their interactions. Because of this, descriptions of this overall reaction network have been advanced using ensembles of ordinary differential equations (ODEs) [2], [3], [4], [5], [6], [7] or more elaborate mathematical constructs for both closed and flow based model systems [8], [9], [10], [11], [12], [13], [14], [15], [16]. Our work has focused on developing and validating an ODE-based description limited to the tissue factor (Tf) pathway to thrombin formation [2] and then using this model in concert with empirical studies to develop concepts of normal [17] and aberrant thrombin generation in individuals and populations with chronic or acute pathologies [18] as well as understanding mechanisms of anticoagulant efficacy [19], [20], [21].

An important issue in developing a predictive model of coagulation with clinical utility is the tension between the complexity of the model (its relative level of congruence with the biological network) and the capacity to measure the actual physiochemical parameters (i.e. initial concentrations of reactants and rate constants) governing the system. With respect to comparatively modeling the coagulation systems of individuals in the human population, the working assumption is that, in the absence of a specific mutation that alters the function of a key enzyme or substrate (*e.g.* factor (f)V Leiden), the rate constants are invariant. Thus measurement error in rate constants would be the primary source of uncertainty in their values [22]. In contrast, the concept of initial species levels is complicated by issues beyond measurement uncertainty, including a lack of information or reasonable assessment methods concerning the *in vivo* concentrations (or surface level expression) of cellular components of the coagulation proteome and the fact that individuals are known to vary in concentrations of soluble coagulation factor precursors. A reasonable resolution of the conflict between model complexity and required input data is a precondition if one is aiming to develop a model that provides therapeutic guidance on an individual basis.

Our approach for modeling individuals has generally been to limit the description of the network to seven circulating precursor proteins (factors II, V, VII, VIIa, VIII, IX, X) and two inhibitors (antithrombin (AT), and tissue factor pathway inhibitor (TFPI)). The rationale for this has four parts: 1) The magnitude of the normal range variation of these soluble proteins between individuals is greater than the measurement uncertainty for these proteins, a methodologic precondition for their use to discriminate among individuals; 2) These proteins appear to be central to the process of Tf initiated thrombin formation [23] and its regulation by anticoagulant agents. Absolute deficiencies in any of these are either incompatible with life [24], or result in bleeding disorders (fV, fX, prothombin, fVIII, fIX or thrombosis (AT) [24], [25]. Additionally, the importance of the four vitamin K dependent (VKD) proteins (fII, fVII/fVIIa, fIX and fX) to normal hemorrhage control is exemplified by their status as primary targets for the anticoagulants warfarin [26] and unfractionated heparin (UFH) [27], both of which have been used for over 60 years. These two therapeutic agents mirror each other in the scope of their action, since UFH potentiates the inhibition of all of the procoagulant enzymes that warfarin anticoagulation targets indirectly by suppressing the levels of their functional precursors; 3) The mathematical representation of the interactions of these proteins in the reaction network appears valid, based on the congruence between empirical reconstructions of this limited network and model descriptions [2], [20]; and 4) Availability of populations with the necessary composition data. Even with the small subset of factors we used in this model, databases containing these 8 measured values for individuals are scarce.

In this study, we systematically perturb, within the acceptable healthy clinical laboratory range, the initial protein concentrations in our ODE based model of Tf-initiated blood coagulation to evaluate the effect on thrombin generation. A unique graphical method is developed to integrate standard measures used to characterize thrombin generation in empirical and computational models (e.g max rate, max level, total thrombin, time to 2 nM thrombin: “clot time”) to visualize how normal range variation in coagulation factors results in unique thrombin generation phenotypes. Three approaches are taken: 1) characterizing the possible range of thrombin generation phenotypes as a function of normal range variation in factor levels, *i.e.* defining the theoretical population range of the healthy coagulant response to Tf; 2) relating the thrombin generation profiles of apparently healthy and hemostatically challenged populations derived using their actual plasma coagulation factor composition to the theoretical “normal” population range; and 3) systematically analyzing the sensitivity of model output of all species collectively and of thrombin specifically to normal range variation in each coagulation factor.

Key findings of these analyses include that normal range variation of coagulation factors yields thrombin generation phenotypes indistinguishable from individuals with some but not all coagulopathies and that coordinate variation of certain pairs of factors disproportionately results in extreme thrombin generation phenotypes, implying that measurement of a smaller set of factors may be sufficient to identify individuals with aberrant thrombin generation potential. These novel types of analyses can ultimately be used to develop clinical tools to evaluate individual procoagulant potential.

## Materials and Methods

### Ethics

Participation of both the individuals undergoing warfarin therapy and the apparently healthy control individuals was approved by the Jagiellonian University Ethical Committee. All participants gave informed written consent.

Participation of severe hemophilia A individuals was approved by the Institutional Review Board at the Centre Hospitalier Universitaire Sainte-Justine Montréal Quebec and approved by the University of Vermont Human Studies Committee. Informed written consent was obtained from all individuals.

### Mathematical Model

The current mathematical model of coagulation utilizes reactions described in publications by Hockin *et al.* [2] and Butenas *et al.* [28] describing the dynamics of tissue factor (Tf) initiated blood coagulation. Inputs to the model include the concentrations of procoagulant factors II, V, VII/VIIa, VIII, IX, X and the anticoagulants TFPI and AT and the rate constants derived from experimental measurements made under conditions of saturating concentrations of phospholipids [2]. The starting concentration of fVIIa was always 1% of the starting fVII concentration. MatLabs stiff solver ode15 s [29] was used to integrate the ODE model with variable time steps whose maximum size was set to 5×10^−3^ s. The model is initiated by exposing the inputs to 5 pM Tf and yields concentration versus time profiles for all of the 34 species representing reactants, intermediates or products (Table S1).

### Populations

Thirty-two apparently healthy individuals recruited from hospital and university staff (Jagiellonian University Medical College, Krakow, Poland) served as controls. Warfarin treated individuals (N = 65; 23 females, 42 males; age: 25–75 years) were apparently on stable anticoagulation (mean time of 4 months; 2≤INR≤3.3). Indications for vitamin K antagonist administration were atrial fibrillation (N = 26), venous thromboembolism (N = 25) or aortic prosthetic valve implantation (N = 14). The exclusion criteria were recent (preceding 6 months) thromboembolic event, acute infection, liver injury, renal insufficiency, autoimmune disorders or known cancer. Three individuals had a thrombotic event subsequent to the blood draw for compositional analysis. Severe hemophilia A individuals (by diagnosis; N = 16) displayed fVIII levels ranging from not detectable to ≤1% at the time of the blood draw used for compositional analysis.

### Modeling thrombin generation in individuals

#### Thrombin generation phenotypes

**Figure 1:**
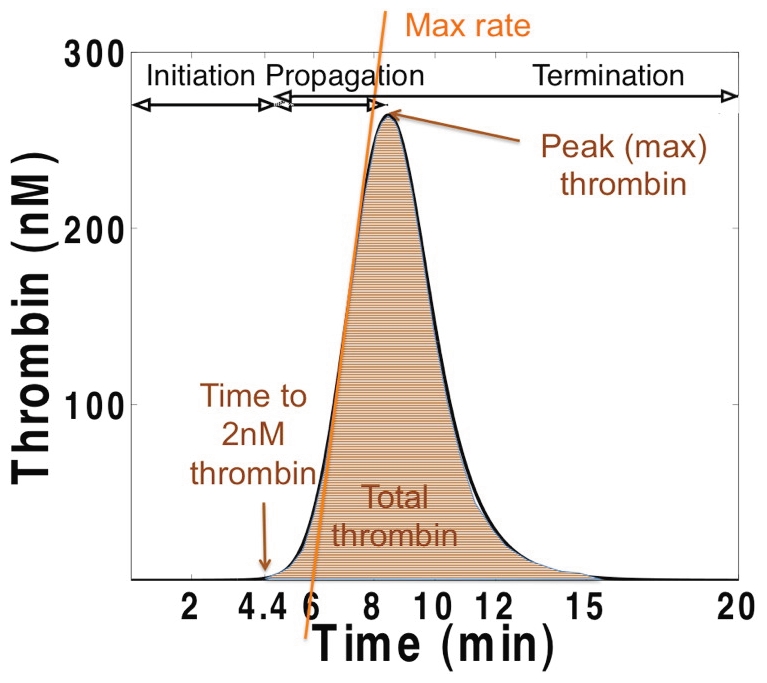
Thrombin generation profile reflecting the dynamics observed in a closed model system. A computationally-simulated time course of thrombin generation with all factors at their mean physiologic level and a 5 pM tissue factor stimulus is shown. Also indicated are the thrombin parameters (time to 2 nM thrombin (clot-time), total thrombin, maximum thrombin, maximum rate) used in this analysis.

In empirical models, thrombin is the most common analyte both because of its ease of measurement and its central and diverse roles [1]. Thrombin generation in these closed model systems displays three distinct phases: *initiation* of coagulation, *propagation* of α-thrombin formation, and *termination* of the procoagulant response (Figure 1). Computationally simulated thrombin generation profiles (cTGPs) were evaluated by standard summary measures that described each curve including the maximum level and rate of thrombin generation, total thrombin generated (the area under the curve) and the time to 2 nM α-thrombin, which corresponds to clot time in our empirical studies [17]. Collectively these 4 parameters are used to define a thrombin generation phenotype.

#### Thrombin generation in a hypothetical normal population

To produce a representation of the distribution of possible thrombin generation phenotypes, the eight factors with non- zero initial concentrations were varied across their normal range. This population of factor ensembles was produced by allowing each factor to have three possible normal range values: extreme low, mean physiologic (factors at 100%) and extreme high (Table S2), yielding (3^8^) permutations. From each of these cTGPs the four thrombin parameters were extracted.

#### Thrombin generation in actual populations

Thrombin generation using actual plasma factor composition data from an apparent healthy population (N = 32), a severe hemophilia A population (N = 16), and a warfarin treated group (N = 65) was simulated and the 4 thrombin parameters extracted for each person in each group. The mean factor levels for each population are presented in Table S3. All factor levels in these populations are within their normal ranges, with the exception of fVIII in the hemophiliac population, and the vitamin K dependent proteins in the individuals undergoing warfarin therapy.

#### Thrombin generation in a hypothetical abnormal population

To produce populations characterized by fIX deficiency, prothrombin deficiency and AT deficiency, the plasma composition data from the apparently healthy population (N = 32) was altered as follows: each individual's fIX level set to 0.01% mean physiologic; or each individual's PT concentration set to 10% (severe PT deficiency) or 40% mean physiologic; or each individual's AT concentration set to 40% (heterozygous AT deficiency) mean physiologic. In each instance, all other factor concentrations were left at their individual measured values.

### Model sensitivity to normal range variation in factor levels

#### Analysis of single factor dependence

To characterize the impact of normal range variation in factor levels on model output of all species, all species with non-zero values at time zero except Tf (8 independent species in total: fII, fV, fVII/VIIa, fVIII, fIX, fX, TFPI, AT) were altered, one at a time, in eleven evenly spaced intervals between the low normal and high normal value for that factor and time course profiles for all 34 species collected. The clinically accepted normal range values were obtained from Fletcher Allen Health Care (Burlington, VT; Table S2). For each of the 8 factors, the collection of cTGPs derived from the 11 initial factor concentrations for a given output species is referred to as the ensemble range for that species with respect to that factor (272 ensembles in total), with the profile reflecting all factors at one hundred percent their mean physiologic value defined as the standard profile for that species.

#### Ensemble standard deviation

In order to evaluate the impact of normal range variation in each factor (*g*) on model output of each species, we utilized a modification of our previously described approach for analyzing our model's sensitivity to perturbations in its rate constants [22]. For any given model output species 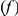 at any selected time (*t*) an ensemble standard deviation (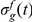) can be calculated. It is designed to represent the variation in that species concentration at time t that occurs as a result of variation in the initial concentration of factor *g* when all other factors are held at their mean physiologic values. A group of predicted time courses (11 time courses) for species (*f*) generated by varying the initial concentration of factor g in linearly spaced intervals across its normal range provides the data set from which the ensemble standard deviation is calculated at 1 second intervals over the 1200 s time course (Figure S1 A & B).

#### Coefficient of variation

The impact of variation in reaction concentration of the each of the 8 initially nonzero factors (*g*) on the production of any model species (*f*) was normalized using a coefficient of variation (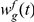) defined to be the ensemble standard deviation at each time t expressed as a fraction of the peak value (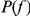) of that species when all factors are initially at 100% of their mean physiologic value (standard model curve). For example, thrombin (IIa) response to normal range variation in TFPI is given by 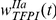 = 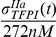, where 272 nM thrombin is the peak concentration of thrombin under standard conditions (see Figure 1). Normalization was performed in order to avoid numerical effects related to the differences in concentrations (>10^6^) between species in the pathway. The peak concentration (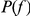) was chosen rather than the corresponding concentration at time *t* from the standard model curve or the ensemble mean curve because these are both time-dependent (Figure S1C).

#### Time averaged coefficients of variation for thrombin

For each of the 8 non-zero initial factors (*g*), the coefficients of variation (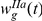) were averaged over the 1200 s time course to yield 8 time averaged coefficients of variation for thrombin (Figure S1C). These 8 values were summed, each individual value expressed as a fraction of that sum, and then ranked by the magnitude of its contribution to the total variation in thrombin induced by normal range variation of the 8 factors.

#### Time averaged coefficients of variation for all model species

For each non-zero factor (*g*) at time (t), the mean coefficient of variation for all resulting protein species is given by
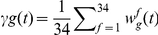
. The time average of 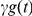 for factor (*g*) over the 20 minutes of the simulation is denoted by 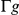. The 8 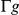 values were summed, each expressed as a fraction of the total, and then ranked by the magnitude of their contribution.

#### Analysis of pair wise variation in factor levels on thrombin generation

Each pair of factors (28 possible) was varied together with the same 11 linearly spaced values within their individual normal ranges, leaving the other 6 factors at their mean physiologic value. This resulted in 121 cTGPs for each factor pair from which the four thrombin parameters were extracted. The range in each of the four parameters induced by variation in that factor pair was then identified and each of these range values expressed as a fraction of the largest perturbation in that parameter observed among the 28 factor pairs. For example coupled variations in AT and TFPI yielded the largest range in the time to 2 nM active thrombin values (a 5 min range between 3 and 8 min) and thus all 27 other ranges for this thrombin parameter are ratioed to this range value.

## Results

The normal range variation in plasma concentration that characterizes the 8 model species with initial non-zero values is presented in Table S2. In order to assess the consequences of this variation, computationally simulated thrombin generation profiles (cTGPs) were produced by assigning a specific normal range value to each of these factors and a constant concentration (5 pM) for tissue factor. In this analysis, the term “individual” refers to a unique ensemble of these 8 factors from which a cTGP, representing the model integrated effect of this ensemble, is generated. The ensemble having all factors at their mean physiologic level serves as a reference cTGP for assessing the relative intensity of thrombin generation characterizing other ensembles. To capture the maximum potential distribution (scope) of cTGPs resulting from normal range variation in these factors, a theoretical population of “normal” individuals, each with a unique ensemble of initial factor concentrations, was generated by allowing each factor to have 3 possible values spanning its normal range (3^8^ or 6561 individuals). To quantify differences between these cTGPs, thrombin parameters were extracted from each cTGP (see Figure 1).

### Factor composition and thrombin generation phenotypes

**Figure 2:**
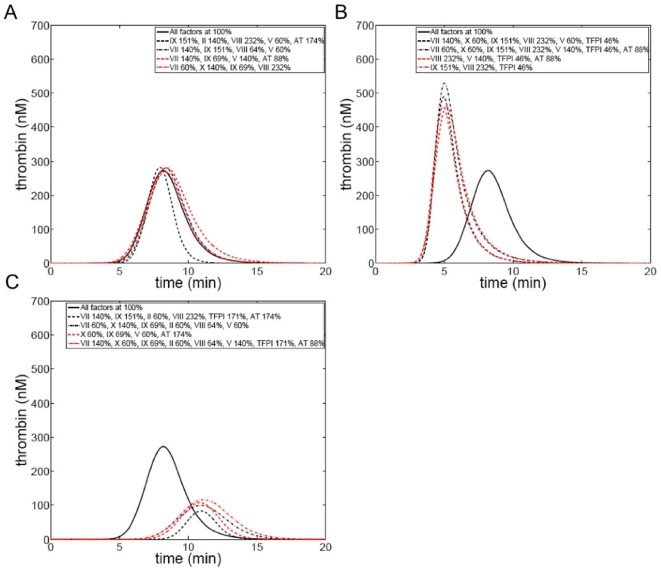
Thrombin generation time courses from selected individuals from an hypothetical population defined by normal range variation in factors. Individuals were selected with disparate factor composition, but similar thrombin generation profiles and compared to an individual (control) with all factor levels at their mean physiologic value. Insets detail factor composition (as percentage of mean physiologic) which is variable among these individuals, with all other factors that are not listed at mean physiologic values. Panel A: 4 individuals similar to the control profile; Panel B: 4 individuals with accelerated and more robust thrombin generation relative to the control; and Panel C: 4 individuals with suppressed and delayed thrombin generation relative to the control.

Figure 2 presents cTGPs of groups of individuals in the theoretical population selected because their cTGPs showed significant overlap despite their disparate factor composition. Factor ensembles (presented in the figure insets) with ∼50% or greater differences in 4 to 8 factor concentrations characterize these individuals. Such individuals, representing disparate factor ensembles but with similar cTGPs, are defined to have the same thrombin generation phenotype. Thus three thrombin generation phenotypes are represented in Figure 2.

In panel A, individuals with cTGPs that overlap the reference cTGP are shown. Panels B and C show individuals with similarly disparate factor composition but overlapping cTGPs that display more or less robust thrombin generation respectively. In panel B, normal range factor variation produces ensembles resulting in cTGPs displaying a 2–3 fold shortening of the clot time parameter and 2 to 3 fold increases in the parameters max rate, max level and total thrombin compared to the reference cTGP. Panel C displays distinct ensembles that produce overlapping cTGPs characterized by a relatively attenuated response: a 2–3 fold prolongation of the clot time parameter and 2 to 3 fold decreases in the parameters max rate, max level and total thrombin compared to the reference cTGP.

The results of these limited comparisons highlight a consequence of normal range variation in factor levels on thrombin generation: factor variation per se (analyzing factor concentrations and not their integrated effect) is not a sufficient discriminator for predicting differences in thrombin generation between individuals. Ensembles, when integrated mechanistically, can effectively compensate for apparently procoagulant or anticoagulant variations in individual factor levels [18], yielding similar thrombin outputs.

### The possible range of “normal” thrombin generation phenotypes

**Figure 3:**
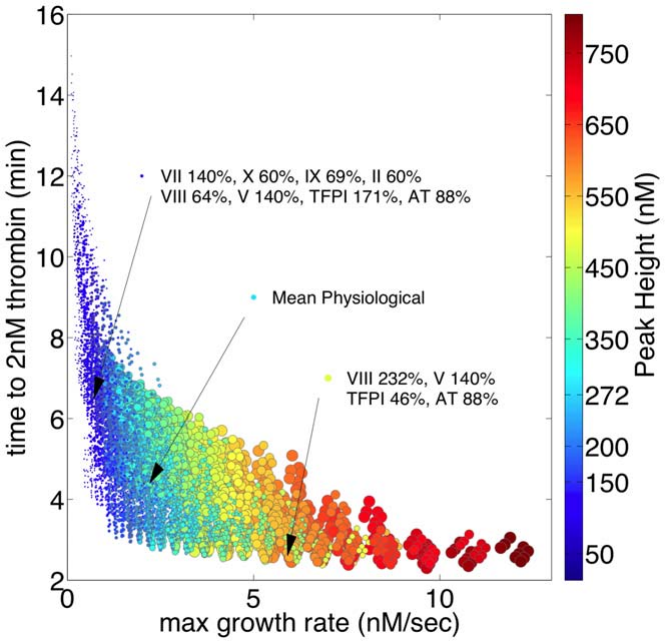
Thrombin generation phenotypes in an hypothetical population defined by normal range variation in factor levels. Each individual in the population (6,561) is defined by 4 thrombin parameters and their phenotype represented graphically by a positioned colored circle: y axis—time to 2 nM thrombin, range (2.3→15 min); x axis—maximum rate of thrombin generation, range (0.1→12.4 nM/s); color—maximum thrombin level, range (23 (dark blue)→792 nM(brown)); and size—total thrombin, range (8,179•→134,340 sec•nM ○). Inset: An individual with all factors at their mean physiologic value is depicted, the arrow indicating that individual's position in the population. Similarly, representative individuals from Figure 2 panels B and C are included.

Figure 3 compares all individuals in the theoretical population in terms of their relative ability to generate thrombin by creating a graphic representation of each individual that reflects the magnitude their thrombin parameters. Individuals (model integrated factor ensembles) are depicted by a positioned, colored ball of specific size, a collective representation of the four thrombin parameters extracted from their respective cTGPs. Time to clot (y axis) and max rate parameters (x axis) position each individual, while color indicates the max level and size defines the total thrombin parameter. To relate the differences between cTGPs observed in Figure 2 to this form of presentation, three individuals are highlighted: an individual with all factors at mean physiologic concentrations and individuals from Figures 2B and 2C. The levels of variation for the thrombin parameters in this population are as follows: 6.5 fold for the clot time (2.3 to 14.97 min); 33.4 fold for max level (23.7 to 792.4 nM); 120 fold for max rate (0.1 to 12.4 nM/s); and 17 fold for total thrombin (8,179 to 134,338 nM•s) (see Table S4). Thrombin parameters for the individual with all factors at mean physiologic values are: clot time—4.4 min; max rate—2.21 nM/s; max level—271.4 nM; and total thrombin—56,458 nM•s.

This population is designed to set the outer boundaries for the types of thrombin generation phenotypes possible because of normal range variations in coagulation factor levels. As is evident from Figure 2 and visual inspection of Figure 3, significant overlap of individuals occurs, and thus the number of thrombin generation phenotypes is less than the number of individuals (factor ensembles). The question that presents itself is whether all potential phenotypes derived from ensembles with normal factor levels are representative of a normal or healthy hemostatic response?

### Normal thrombin generation phenotypes; possible range vs actual

Computationally analyzed thrombin generation using factor composition data from an apparently healthy control group of 473 individuals from the Leiden Thrombophilia Study has been reported [17]. Factor level variation in this population was similar to or exceeded the ranges used to generate the theoretical population presented in Figure 3 (Table S2). In this population of Dutch individuals (272 women, 201 men) the range in thrombin parameters was: 3.3 fold for the clot time; 3.9 fold for maximum level thrombin; 4.8 fold for maximum rate; and 4.5 fold for total thrombin. The 2 to 20 fold larger ranges predicted for the thrombin parameters of the theoretical population reflect factor ensembles that were possible in the LETS population (given the factor composition ranges) but that did not occur.

The wider ranges of thrombin parameters characterizing the theoretical population have two potential origins: a methodologic one due to its larger size, emphasis on the extremes of each factor range and its treatment of all possible ensembles as of equal probability; or a biological one reflecting the fact that some ensembles, perhaps those resulting in individuals with the more extreme characteristics in Figure 3, are consistent with coagulopathic states and thus would not be found in a healthy population.

Relevant coagulation factor composition data from comparably sized populations of apparently healthy individuals are not available currently. However, factor composition data for smaller populations, including those with coagulopathies resulting from inherited or pharmacologically induced deficiency states, are available. A comparative analysis of individuals with defined, composition-based hemostatic defects resulting in a diminished coagulant response was performed to determine whether their thrombin generation phenotypes fall within the theoretical normal range population distribution.

**Figure 4:**
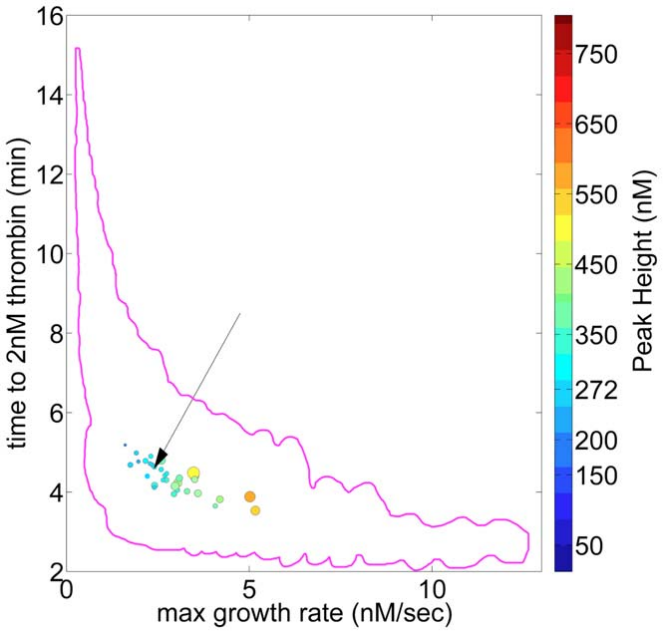
Thrombin generation phenotypes in a population of apparently healthy individuals. Plasma factor composition for 32 individuals was used to generate time courses of thrombin generation, thrombin parameters were extracted and each individual represented as described in the legend to Figure 3. The boundaries (magenta) of the theoretical population are outlined and an individual with all factors at their mean physiologic values is also presented. Arroe indicates an individual with all factors at mean physiological.

Figure 4 presents the graphic representation of the thrombin parameters characterizing a population of apparently healthy individuals (N = 32), with the boundary of the theoretical population (from Figure 3) also shown. Factor level variation in this population is presented in Table S2 and the mean factor levels in Table S3. The max level and max rate parameters vary ∼3 fold in this population, the total thrombin parameter ∼4 fold and the clot time parameter ∼1.4 fold. The parameter ranges for max level, max rate and total thrombin are similar to those reported for the larger LETS population (N = 473) [17] while the range of clot time values in this population is ∼40% that of LETS. Thus both populations appear confined to a relatively small region of the potential distribution of thrombin generation phenotypes available because of normal range variation in coagulation factor levels.

### “Abnormal” thrombin generation phenotypes

**Figure 5:**
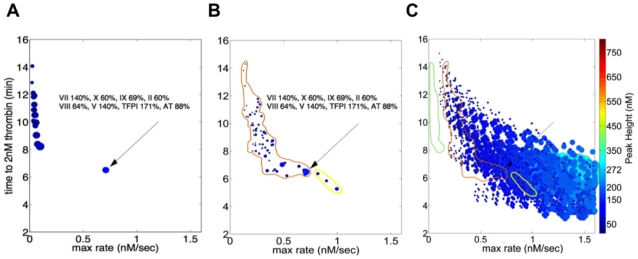
Thrombin generation phenotypes in hemophilia A individuals and individuals undergoing warfarin therapy. Plasma factor composition was used to generate time courses of thrombin generation, thrombin parameters extracted and each individual represented as described in the Figure 3 legend. The x axis (max rate) is truncated (0–1.5 nM/s) and the size of each individual symbol (total thrombin parameter) has been increased by a factor of 5 relative to Figure 3 to improve visibility. Also indicated (arrow) is an individual shown in Figure 2C and Figure 3. Panel A: 16 individuals with severe hemophilia A (fVIII: 0.07% to 1% mean physiologic). Panel B: 65 individuals stablely anticoagulated with warfarin (INR values between 2 and 3.3). The three individuals who subsequently had a thrombotic event are circled in yellow. Panel C: region of the hypothetical population distribution displaying the most similar thrombin generation parameters; the boundaries of the distributions of the hemophilia (green) and warfarin populations (orange+yellow (3 individuals)).

Figure 5 presents a comparison of thrombin generation between a group of severe hemophilia A individuals (N = 16; factor VIII<1%; panel A), a cohort of individuals anticoagulated with warfarin (N = 65; panel B) and the relevant subset of the theoretical population (panel C). Plasma composition data for the hemophilia and warfarin treated populations are presented in Table S3. To facilitate the comparison, the max rate (x axis) parameter extends only to 1.5 nM/s and the size of each individual's symbol (total thrombin parameter) has been increased by a factor of 5 relative to Figure 3 to improve its visibility. The boundaries for the hemophilia and warfarin-treated groups are indicated in panel C.

In general, individuals with severe hemophilia A, in the absence of replacement therapy with rfVIII or other agents, experience prolonged and potentially life threatening bleeding in response to a hemostatic challenge as well as episodes of “spontaneous bleeding” [30]. In this hemophilia population, all factors other than fVIII are within the normal range (Table S3). FVIII concentrations vary from 0.07% to 1% mean physiologic. For the overall population the parameter ranges were: time to 2 nM thrombin—y axis, range (8.2→14 min); maximum rate of thrombin generation—x axis, range (0.02→97 nM/s); maximum thrombin level—color, range (16→50 nM); and total thrombin—size, range (17,300→40,845 sec•nM).

As can be seen by comparing the distribution of phenotypes in panel C with that of panel A, the hemophilia population is positioned outside the most extreme phenotypes in the theoretical normal population. These individuals are characterized by lower max rates but substantially higher total thrombin values across their distribution than their nearest neighbors in the theoretical population. The defect in thrombin generation occasioned by severe fVIII deficiency also segregates these individuals from the warfarin-treated group, again drive by differences in max rate and total thrombin parameters.

The warfarin-treated individuals represented in Figure 5B, were initially considered, in terms of their clinical history, to be stably anticoagulated, as assessed by a 2 to 3 fold prolongation of their plasma clotting time in a standardized assay (INR: 2 to 3.3). In this population, the non-VKD protein concentrations are all within the normal range, while the VKD proteins (fII, fX, fIX, fVII/fVIIa) are suppressed 50 to 90%, with the level of suppression of each VKD protein varying between individuals. For the overall population the parameter ranges were: time to 2 nM thrombin, range (5.3→17 min); maximum rate of thrombin generation, range (0.08→1 nM/s); maximum thrombin level, range (13→100 nM); and total thrombin, range (6,048→18,978 sec•nM).

The 65 individuals of the warfarin-treated population distribute within the region of the theoretical population characterized by low max rates and prolonged clot times (Figure 5A). This is demonstrated more clearly in Figure 5C, where the boundaries of the warfarin treated population are indicated by the orange line. Their overall characteristics, *i.e.* their 4 thrombin parameters, do not distinguish them from their nearest neighbors in the theoretical population, suggesting that this region of the theoretical population is characterized by thrombin generation phenotypes reflecting a compromised coagulant response.

Three of the warfarin-treated individuals (circled in Figure 5B) were subsequently reported to have suffered a thrombotic event. The graphical method employed separates these individuals from the remainder of the warfarin-treated group, primarily because of their max rate parameter, consistent with the idea that they were under anticoagulated despite clinical INR values between 2.1 and 2.5. Inspection of the plasma factor composition data for these three individuals shows at most minor differences between their VKD protein levels (fII 30±3%; fVII 35±6%; fIX 42±2%; fX 32±13%; mean±SD) and the overall warfarin-treated population (Table S3); however, within the non VKD proteins, their TFPI values (74±3%, mean±SD) are at the low end of the range characterizing this population while their fVIII values (212±7%, mean±SD) are at the high end (Table S3). These compositional data are consistent with the graphical characterization of these individuals as being under anticoagulated compared to the whole group in two ways: the pattern of high fVIII and low TFPI levels is computationally consistent with more robust thrombin generation; and the prothrombin time assay, which is the basis for INR metric, is relatively insensitive to variations in TFPI and FVIII levels and thus would not identify these individuals as insufficiently anticoagulated.

To further test the “normalcy” of our theoretical population of thrombin phenotypes, additional populations representing “bleeding” phenotypes (fIX deficiency, prothrombin deficiency) or prothrombotic phenotypes (antithrombin deficiency) were analyzed. These populations were generated using the group (N = 32) of apparently healthy individuals for which factor composition data was available (Tables S2 & S3). In each case, all factors were left at their individual specific values except fIX or prothrombin or antithrombin, which were set to an average value characterizing their clinical deficiency state.

**Figure 6:**
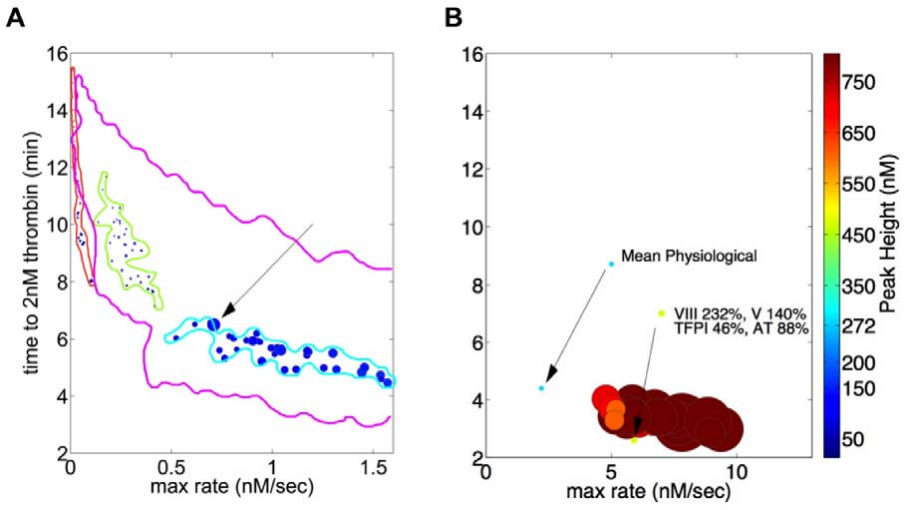
Thrombin generation phenotypes for hypothetical fIX deficiency, fII deficiency and AT deficiency. Plasma factor composition for each of 32 apparently healthy individuals was altered in one factor to reflect each deficiency state, time courses of thrombin generation analyzed for thrombin parameters and each individual represented as described in the Figure 3 legend. Panel A: severe fIX deficiency (fIX = 0.01% mean physiologic); severe fII deficiency (fII = 10% mean physiologic); heterozygous fII deficiency (fII = 40% mean physiologic). The x axis (max rate) is truncated (0–1.5 nM/s) and the size of each individual symbol (total thrombin parameter) has been increased by a factor of 5 relative to Figure 3 to improve visibility. Also included is the reference individual from Figure 2C. Panel B: heterozygous AT deficiency (AT = 40% mean physiologic). Note that x axis shows the full range depicted in Figures 3 and 4. An individual with all factors at mean physiologic is shown along with an individual in Figure 2B and Figure 3. The boundaries of the hypothetical population are also shown in Panel A.

Figure 6A presents the distributions of the individuals in the artificial fIX and prothrombin deficient groups. The outer boundaries of the theoretical population are depicted by the yellow line, with each group representing a one factor deficiency state circumscribed to define its limits. As with Figure 5, the size of each individual's symbol (total thrombin parameter) has been increased by a factor of five to improve its visibility and the x axis is truncated relative to Figure 3.

The fIX deficient population was modeled to represent a severe hemophilia B state, with fIX levels set to 0.01%. In general the bleeding problems associated with severe fIX deficiency (fIX<1%) are similar to those characterizing hemophilia A [31]. The distribution of this artificial hemophilia B population lies outside the hypothetical population and appears roughly equivalent to the one characterizing actual hemophilia individuals (Figure 5A). Differences in the total thrombin parameter between the actual hemophilia A individuals (Figure 5A) and the artificial fIX deficient individuals reflect the fact that most of the hemophilia A individuals have higher fVIII levels than the fIX level selected for the “hemophilia B” population.

Two levels of prothrombin deficiency are also represented in Figure 6A, with the prothrombin concentration set to 10% or 40% of its mean physiologic value in each of the 32 control individuals. Clinically, prothrombin deficiency is a rare coagulation disorder with homozygous individuals displaying prothrombin levels less than 10% mean physiologic; it is characterized by severe, often life threatening bleeding episodes [32]. Heterozygous individuals with prothombin levels 40 to 60% mean physiologic are usually asymptomatic, with excess bleeding occurring occasionally after surgical procedures.

The model representation of homozygous prothrombin deficiency (Figure 6A) places these individuals along the edge of the theoretical population, overlapping, with respect to three of the thrombin parameters, the more highly anticoagulated individuals in the warfarin population (Figure 5B). However, the total thrombin parameter for individuals with this level of PT deficiency is suppressed relative to the total thrombin values typifying the nearest neighbors in the theoretical population and the warfarin-treated population. The distinction between stably anticoagulated individuals on warfarin and severe prothombin deficiency is consistent with the more extreme hemorrhagic phenotype seen in severe prothrombin deficiency.

In contrast, thrombin parameter analysis of individuals modeled to be heterozygous in their prothombin deficiency (40% mean physiologic, Figure 6A) indicates that this population is embedded within the boundaries of the theoretical population. An individual from Figure 2C is also graphed to provide a comparison to a conventional representation of thrombin generation for this region of the theoretical population. Neither max level nor total thrombin parameters distinguish these individuals from their nearest neighbor in the theoretical population. If one excludes the three warfarin-treated individuals who proved to be insufficiently anticoagulated, these individuals are situated outside the warfarin-treated population, displaying shorter clot times and larger max rates, parameter differences consistent with their overall lack of bleeding incidents. An individual from Figure 2C is also graphed to provide a comparison to a conventional representation of thrombin generation for this region of the theoretical population.

Heterozygous AT deficiency, with an incidence rate of 1 in 500 to 1 in 5000 in the general population, is characterized by AT concentrations 40 to 60% mean physiologic, below the normal range variation of ∼80 to 170% mean physiologic [33](Table S2). These lower levels of AT induce a prothrombotic phenotype associated with a 5 to 50 fold increased risk for venous embolism [34].

The results of altering AT levels in the 32 control individuals to 40% mean physiologic are presented in Figure 6B. The scaling is the same as the theoretical population displayed in Figure 3. As can be seen by visual inspection of Figures 3 and 6B, this level of AT deficiency yields individuals with extreme thrombin generation phenotypes with respect to the parameters max level and total thrombin. Comparison with the model representation (Figure 4) of the same individuals prior to the induction of AT deficiency also shows a systematic increase (∼2 fold) in the max rate parameter. None of the nearest neighbors in the theoretical population (Figure 3) display similar max level and total thrombin parameters. In fact, no individual in the theoretical population displays total thrombin levels of the magnitude characterizing the AT deficient population. The mean total thrombin parameter in the AT deficient group (392,776 nM•s) exceeds that of the matching 32 controls (71,000 nM•s) by ∼5.5 fold.

### Single factor contribution to overall variation in thrombin generation

Table 1 presents the results of an analysis testing the sensitivity of model outputs to normal range variation in the 8 initial nonzero factor levels. Each factor was set sequentially to 11 values spanning its normal range, the other 7 factors held at their mean physiologic values and the time courses for all 34 model output species collected. Analysis (Figure S2) resulted in the generation of time averaged coefficients of variation for all 34 output species which were manipulated ultimately to rank each factor by the magnitude of the contribution its normal range variation makes to variation in all model species or variation in thrombin generation (Figure S3). It is this ranking, the explained variance, which is presented in Table 1.

**Table 1:** Ranking factors by the effect that normal range variation in their initial values has on model output.

A.			B.	
Rank	Factor	IIa Exp Var	Factor	All Factors Exp Var
1	TFPI	32.0	TFPI	30.9
2	II	16.5	VIII	18.2
3	VIII	14.6	AT	12.0
4	AT	12.5	IX	12.0
5	IX	10.8	X	10.7
6	X	5.1	II	7.4
7	V	4.3	V	5.1
8	VII	4.0	VII	3.7

Panel A: The explained variance is defined as the time averaged coefficient of variation for thrombin for a given factor expressed as a fraction of the sum of all the time averaged coefficients of variation for thrombin for the 8 factors. Panel B: For a given factor, the effect of varying its level across its normal range on all model species is defined as the mean of the time averaged coefficient of variations for all 34 species. The explained variance is then defined as fraction of the sum of the mean time averaged coefficients of variation for the 8 factors (**Figures S2 and S3**).

These analyses indicate that two factors account for ∼50% of the observed sensitivity of model output, whether the generation of thrombin is considered or all output species are assessed. Variation in the initial TFPI concentration has the greatest impact on both outputs while variation in the fII level is the second most effective contributor to overall differences in thrombin generation. In general this analysis suggests that TFPI alone or coordinated normal range variation of a few factors may account for the extreme thrombin generation phenotypes in the “normal” theoretical population.

### Factor pair induced variation in thrombin parameters

To further explore the relationship between outlying thrombin generation phenotypes and initial factor composition, a comparison focusing on the effect of normal range variation of pairs of factors was conducted. The effects of factor pair variation were quantified in terms of the magnitude of the range of potential thrombin parameter values induced by the coordinated variation in the concentrations of each pair of factors. Unlike the sensitivity analysis ranking the global effects of single factor variation (Table 1), this approach quantifies the effects of variation on each thrombin parameter, facilitating direct comparisons to the distributions of individuals observed in graphical representations of the various populations.

**Figure 7:**
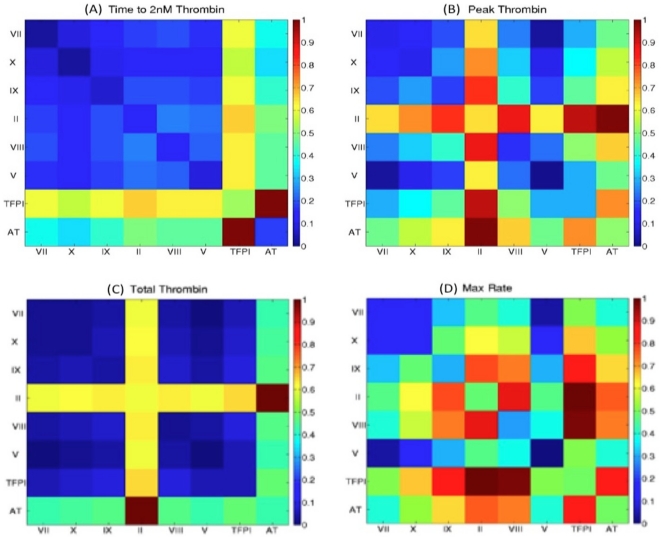
Factor pair induced variation in thrombin parameters. Pairs of factors (28 possible) were varied through their normal range, thrombin parameters were extracted from resulting cTGPs, the factor pair induced ranges for each thrombin parameter established, and then the set of 28 ranges for each thrombin parameter expressed as a function of the largest induced range for that parameter. The color scale reflects the normalized range values. Each parameter box (64 normalized range values) shows the 28 factor pair effects (in duplicate) and the relative intensity of each single factor (8 total) contribution to variation in the indicated thrombin parameter (see reverse diagonal: bottom right to upper left).

Figure 7 presents the results of this analysis, with the color scale reflecting the normalized range values. Each thrombin parameter box displays 64 range comparisons as colored squares: 28 factor pair effects are ranked (in duplicate); and the intensity of each single factor (8 total) contribution to variation in the indicated thrombin parameter is represented in the reverse diagonal: bottom right to upper left. Table 2 presents a summary of the most potent single and factor pair contributors to variation in each thrombin parameter.

**Table 2:** Most potent inducers of alteration in thrombin parameters: single versus factor pair variation.

Thrombin Parameter	Single Factor	Factor Pair
**Clot Time**	TFPI	(TFPI, AT)
**Max Thrombin**	II	(II, AT)>(TFPI, II)
**Max Rate**	AT = TFPI≥II	(TFPI, II) = (TFPI, VIII)
**Total Thrombin**	II>AT	(II, AT)

Visual inspection of Figure 7 highlights the differences between each thrombin parameter's sensitivity to every single factor and factor pair induced variation. Maximum parameter ranges induced by single factor variation are approximately half that induced by the most effective pair for each parameter. Variations in the parameters clot time and total thrombin are dominated by coordinate variation of AT and TFPI and AT and fII respectively, with more than one half the other factors pairs showing relatively minimal effects (<20% of the most effective pair). In contrast, the analysis shows the parameters max rate and max level to have a more complex dependency: strong sensitivity (induced ranges at least 80% of that for the most effective pair) is observed with coordinate variation in 8 factor pairs for the max rate parameter and for 6 factor pairs with the max level parameter.

## Discussion

The concentrations of the components of the coagulation proteome of blood, as measured by standard laboratory tests, vary among apparently healthy individuals, often ranging ±40% to 50% of the mean population value (*e.g.* Table S2). The significance of this variation remains relatively unexplored in part because the imposition of the category of “healthy” implies these differences are background noise and have no hemostatic consequence.

In this study we have attempted to define the consequences of normal range variation of components of the coagulation proteome by using a mechanism based computational approach that translates coagulation factor concentration data into a representation of an individual's thrombin generation potential. Unique ensembles of the 8 coagulation factors used as initial conditions for the computational modeling were taken to represent individuals in a theoretical healthy population and then compared to normal and “abnormal” individuals, *i.e.* factor ensembles measured in apparently healthy individuals, actual coagulopathic individuals or artificially constructed factor ensembles representing individuals with specific factor deficiencies. A sensitivity analysis was then performed to rank either individual factors or all possible pairs of factors in terms of their contribution to the overall distribution of thrombin generation phenotypes.

Although limited by its size, the analysis of actual healthy individuals tentatively indicates that the actual normal distribution is constrained to a fraction of the theoretical range of “normal” phenotypes. Comparison of the theoretical population to individuals with a hemorrhagic phenotype shows that normal range variation cannot generate low thrombin generation phenotypes as extreme as those seen in severe hemophila A or B. Thus the overt hemorrhagic problems seen in affected individuals would not be a predicted outcome of normal range variation. Similarly the extreme high thrombin generation phenotype associated with AT deficiency is not reproduced by normal range variation, potentially suggesting a limit to the severity of the thrombotic risk associated normal range variation. However, such variation does yield some thrombin generation profiles that are “abnormal”, *i.e.* the same as phenotypes characterizing individuals with other less severe composition-based coagulopathies, *e.g.* that induced by warfarin anticoagulation. Collectively the data suggest that unremarkable composition data from a standard laboratory screen of coagulation factors is not an absolute guarantee of a properly calibrated response to vascular injury. Composition based analyses of larger cohorts of apparently healthy as well as hemostatically challenged individuals, especially those with thrombotic phenotypes, will be necessary to better establish the boundaries of “normal” thrombin generation.

The sensitivity analysis assessing the effect of pairwise variation of coagulation factor concentrations identifies the two inhibitors in the network, TFPI and AT, as potent inducers of overall variation (Table 2, Table S4). Coordinate expression of extreme high normal range TFPI and AT concentrations is sufficient to yield phenotypes similar to individuals characterized by impaired thrombin generation, *i.e.* prolonged clot times, and lower max rate, peak and total thrombin values; this effect is amplified when fVIII levels are simultaneously at the low end of their normal range (Table S4) Identifying factors to which the thrombin output is least sensitive (fVII, fV and fX in this analysis) to their normal range variation, singly or when assessed paired with other factors, could reduce the number of input analytes required to capture the important features of each individual response to injury.

The assessment of the potential of an individual's blood or derived plasma fraction to generate thrombin has and continues to be the primary method of hemostatic monitoring; defects in thrombin generation are identified by relative assay performance differences comparing an individual's outcome to an outcome typical of apparently healthy individuals. Historically these assays are designed to monitor clot time as the indicator of hemostatic competence and are most sensitive to gross differences in composition, e.g. severe deficiencies of specific factors [25]. More recently “global” thrombin assays have provided a more robust account of the flux of thrombin generation in closed systems after tissue factor initiation and their applicability to the diagnosis of coagulopathies is an area of active research [35], [36], [37], [38], [39], [40], [41]. However, as with the clot based assays, those readouts, whether defined as typical or atypical, do not explain the origins of their features and as to why one individual appears the same or different from another. This modeling based approach requires coagulation factor analyses of each individual's citrate plasma sample, but yields a representation of an individual's coagulation state that is easy to dissect, based on current understanding of the dynamics reflecting proteins at their physiologic concentrations and native conformations. It creates a mechanism-based rationale for asking the question as to whether individuals can be relatively closer to a hemorrhagic or thrombotic problem and how composition changes in a subset factors driven by other disease processes, *e.g.* inflammatory syndromes, might have different hemostatic consequences in different individuals.

## Supporting Information

Figure S1
**Sensitivity of a model species (α-thrombin) to variation in initial factor concentration.** Thrombin generation profiles resulting from varying in eleven intervals the initial concentrations of TFPI (panel A: 46–171%) or AT (panel B: 88–171%) across their normal range (Low: dotted, high: dash-dot, and 100%: dashed curves) are shown. The solid bold lines in these panels represent the ensemble standard deviation associated with the mean thrombin concentration at each time point. Panel C: The coefficient of variation (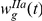) at each time point is plotted for TFPI and AT. The time averaged coefficient of variation values are shown in the parentheses and represent the mean of the coefficient of variation values across the 20-min simulation.(PDF)

Figure S2
**Thrombin sensitivity across the normal range for each non-zero factor (g) at selected times.** Coefficient of variation for thrombin (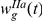) characterizing predicted thrombin concentrations is plotted for each of the 8 protein factors at reference times (Figure 1) during the coagulation process. In panels representing 2.0 & 20.0 min, insets shows changes in the coefficient of variation that are dramatically smaller than other time points (10-4). Large bars imply that normal range variation leads to relatively higher variability in the level of thrombin at that time point.(PDF)

Figure S3 **Aggregate sensitivity of model species as a function of normal range variation of each factor (** ***g*** **) at selected times.** Coefficients of variation for the 15 most sensitive model species for each of the 8 non-zero protein factors (*g*) at relevant times during the coagulation cascade are presented. Each species coefficient of variation is represented by a color and its magnitude by the length. Long bars imply the greatest effects of normal range variation on the dynamics of the simulation.
(PDF)

Table S1 **Ordinary differential equations comprising the model.**
(PDF)

Table S2 **Typical initial coagulation factor concentrations and their normal ranges.**
(PDF)

Table S3 **Factor levels for control, hemophilia & warfarin groups (Mean ± SD).**
(PDF)

Table S4 **Hypothetical normal range plasma compositions resulting in extreme thrombin generation phenotypes.** A rank ordering for each of the 6561 simulations for four metrics is shown with the combination of initial factor concentrations that produced them.
(PDF)
