# Supplementary figures and images for: Defining the Boundaries of Normal Thrombin Generation: Investigations into Hemostasis
Source: PLoS One. 2012 Feb 2;7(2):e30385. doi: 10.1371/journal.pone.0030385 (PMC3271084; doi:10.1371/journal.pone.0030385)

**FIGURE S1.**

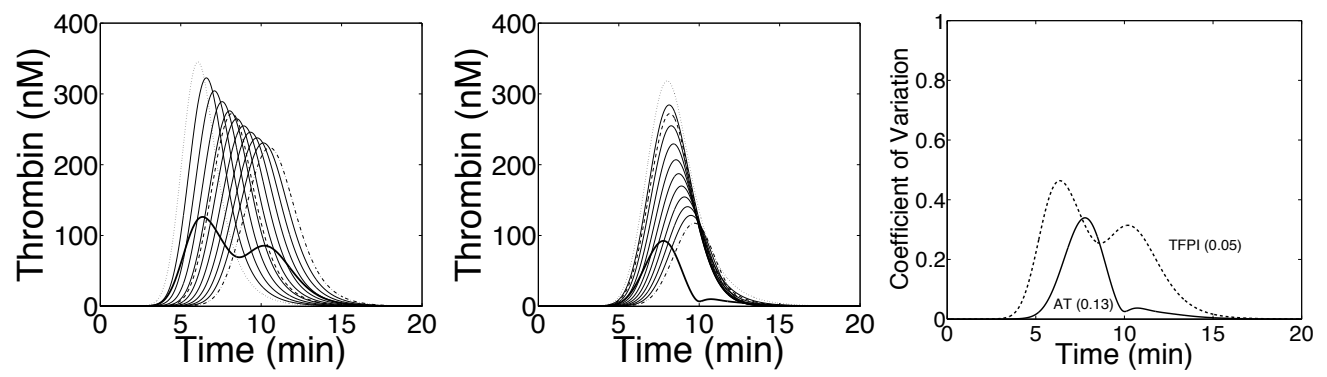

Supplement: Figure S1 — Sensitivity of a model species (α-thrombin) to variation in initial factor concentration. Thrombin generation profiles resulting from varying in eleven intervals the initial concentrations of TFPI (panel A: 46–171%) or AT (panel B: 88–171%) across their normal range (Low: dotted, high: dash-dot, and 100%: dashed curves) are shown. The solid bold lines in these panels represent the ensemble standard deviation associated with the mean thrombin concentration at each time point. Panel C: The coefficient of variation () at each time point is plotted for TFPI and AT. The time averaged coefficient of variation values are shown in the parentheses and represent the mean of the coefficient of variation values across the 20-min simulation. (PDF) [file pone.0030385.s001.pdf]

FIGURE S2.

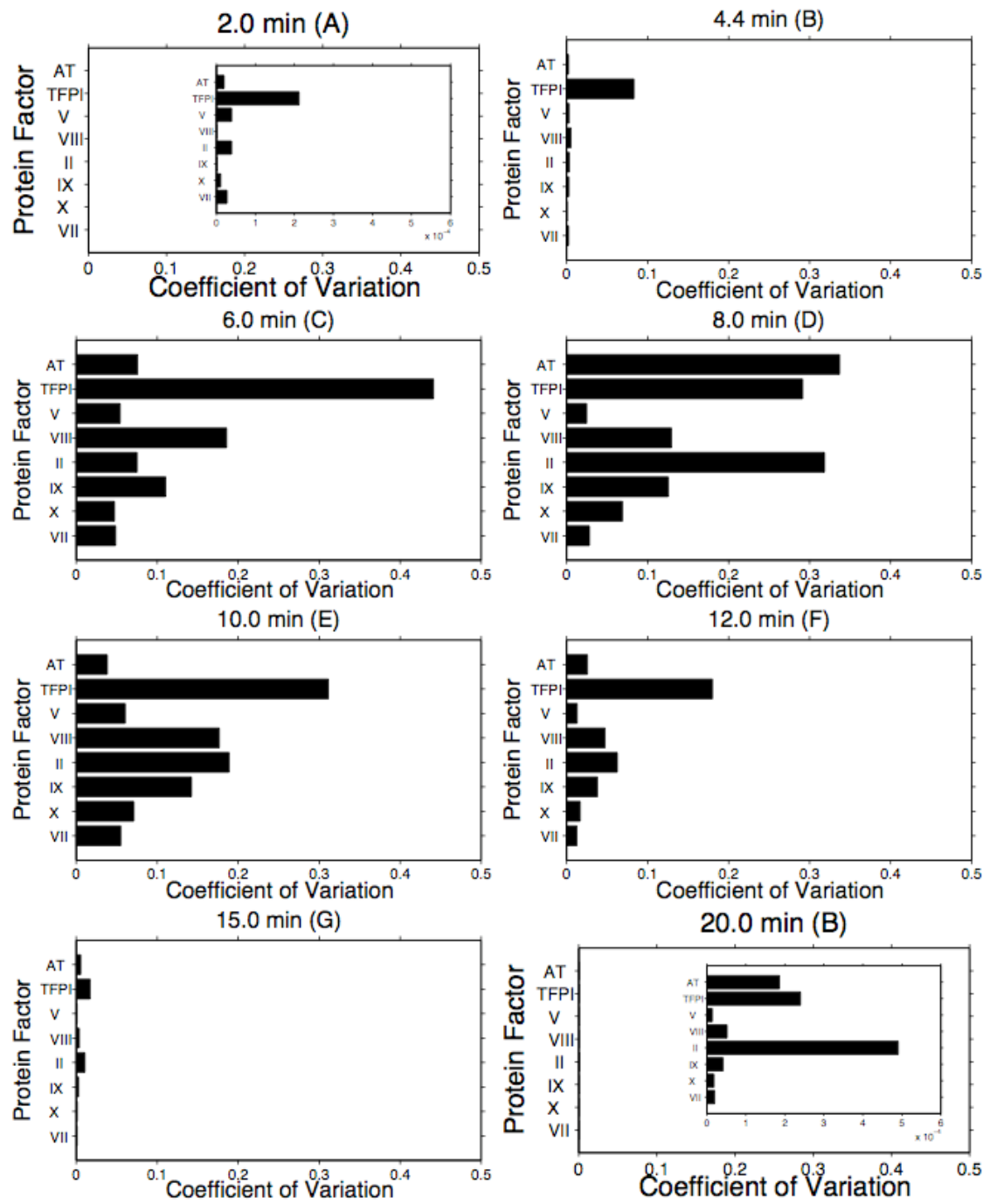

Supplement: Figure S2 — Thrombin sensitivity across the normal range for each non-zero factor (g) at selected times. Coefficient of variation for thrombin () characterizing predicted thrombin concentrations is plotted for each of the 8 protein factors at reference times (Figure 1) during the coagulation process. In panels representing 2.0 & 20.0 min, insets shows changes in the coefficient of variation that are dramatically smaller than other time points (10-4). Large bars imply that normal range variation leads to relatively higher variability in the level of thrombin at that time point. (PDF) [file pone.0030385.s002.pdf]

FIGURE S3.

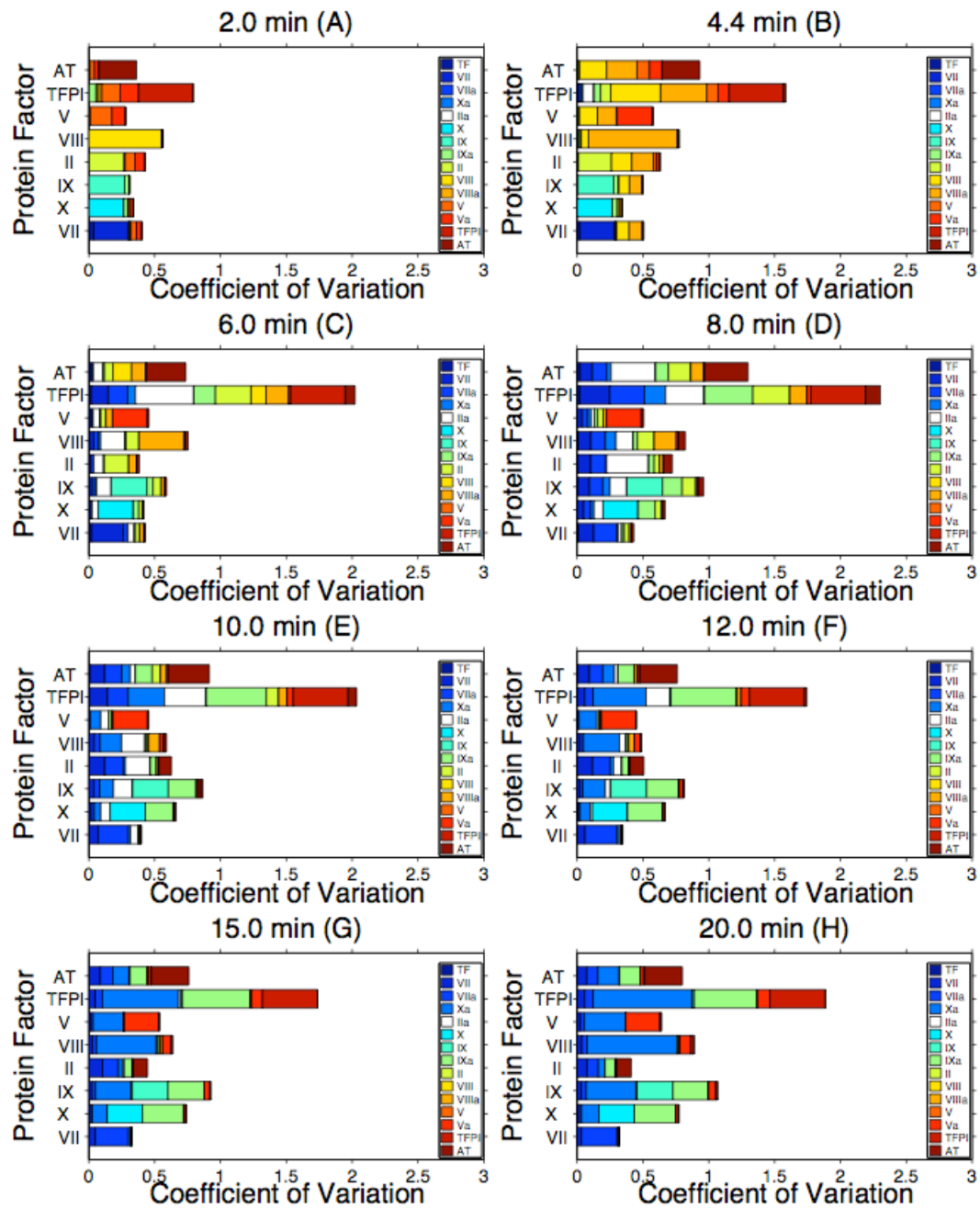

Supplement: Figure S3 — Aggregate sensitivity of model species as a function of normal range variation of each factor ( g ) at selected times. Coefficients of variation for the 15 most sensitive model species for each of the 8 non-zero protein factors (g) at relevant times during the coagulation cascade are presented. Each species coefficient of variation is represented by a color and its magnitude by the length. Long bars imply the greatest effects of normal range variation on the dynamics of the simulation. (PDF) [file pone.0030385.s003.pdf]
