# Supplementary material for: Defining the Boundaries of Normal Thrombin Generation: Investigations into Hemostasis
Source: PLoS One. 2012 Feb 2;7(2):e30385. doi: 10.1371/journal.pone.0030385 (PMC3271084; doi:10.1371/journal.pone.0030385)
Supplement: Table S1 — Ordinary differential equations comprising the model. (PDF) [file pone.0030385.s004.pdf]

SUPPLEMENT

TABLE S1: Ordinary differential equations comprising the model.

$$\frac{d[TF]}{dt} = -k_2[TF][VII] + k_1[TF=VII] - k_4[TF][VIIa] + k_3[TF=VIIa] \tag{1}$$

$$\frac{d[VII]}{dt} = -k_2[TF][VII] + k_1[TF=VII] - k_5[TF=VIIa][VII] - k_6[Xa][VII] - k_7[IIa][VII] \tag{2}$$

$$\frac{d[TF=VII]}{dt} = -k_1[TF=VII] + k_2[TF][VII] \tag{3}$$

$$\frac{d[VIIa]}{dt} = -k_4[TF][VIIa] + k_3[TF=VIIa] + k_5[TF=VIIa][VII] + k_6[Xa][VII] + k_7[IIa][VII] \tag{4}$$

$$\begin{aligned}\frac{d[TF=VIIa]}{dt} &= -k_3[TF=VIIa] + k_4[TF][VIIa] - k_9[TF=VIIa][X] + k_8[TF=VIIa=X] - k_{12}[TF=VIIa][Xa] + k_{11}[TF=VIIa=Xa] \\ &\quad - k_{14}[TF=VIIa][IX] + k_{13}[TF=VIIa=IX] + k_{15}[TF=VIIa=IX] - k_{37}[TF=VIIa][Xa=TFPI] - k_{42}[TF=VIIa][AT]\end{aligned} \tag{5}$$

$$\begin{aligned}\frac{d[Xa]}{dt} &= -k_{12}[TF=VIIa][Xa] + k_{11}[TF=VIIa=Xa] + k_{22}[IXa=VIIIa=X] - k_{28}[Xa][Va] + k_{27}[Xa=Va] - k_{34}[Xa][TFPI] \\ &\quad + k_{33}[Xa=TFPI] - k_{38}[Xa][AT] + k_{43}[IXa][X]\end{aligned} \tag{6}$$

$$\frac{d[IIa]}{dt} = k_{16}[Xa][II] + k_{32}[mIIa][Xa=Va] - k_{41}[IIa][AT] \tag{7}$$

$$\frac{d[X]}{dt} = -k_9[TF=VIIa][X] + k_8[TF=VIIa=X] - k_{21}[IXa=VIIIa][X] + k_{20}[IXa=VIIIa=X] + k_{25}[IXa=VIIIa=X] - k_{43}[IXa][X] \tag{8}$$

$$\frac{d[TF=VIIa=X]}{dt} = k_9[TF=VIIa][X] - k_{10}[TF=VIIa=X] - k_8[TF=VIIa=X] \tag{9}$$

$$\frac{d[TF=VIIa=Xa]}{dt} = k_{10}[TF=VIIa=X] + k_{12}[TF=VIIa][Xa] - k_{11}[TF=VIIa=Xa] - k_{36}[TF=VIIa=Xa][TFPI] + k_{35}[TF=VIIa=Xa=TFPI] \tag{10}$$

$$\frac{d[IX]}{dt} = -k_{14}[TF=VIIa][IX] + k_{13}[TF=VIIa=IX] \tag{11}$$

$$\frac{d[TF=VIIa=IX]}{dt} = k_{14}[TF=VIIa][IX] - k_{13}[TF=VIIa=IX] - k_{15}[TF=VIIa=IX] \tag{12}$$

$$\frac{d[IXa]}{dt} = k_{15}[TF=VIIa=IX] - k_{19}[VIIIa][IXa] + k_{18}[IXa=VIIIa] + k_{25}[IXa=VIIIa=X] + k_{25}[IXa=VIIIa] - k_{40}[IXa][AT] \tag{13}$$

$$\frac{d[II]}{dt} = -k_{16}[Xa][II] - k_{30}[Xa=Va][II] + k_{29}[Xa=Va=II] \tag{14}$$

$$\frac{d[VIII]}{dt} = -k_{17}[IIa][VIII] \tag{15}$$

$$\frac{d[VIIIa]}{dt} = k_{17}[IIa][VIII] - k_{19}[VIIIa][IXa] + k_{18}[IXa=VIIIa] - k_{24}[VIIIa] + k_{23}[VIII.lca1][VIII.a2] \tag{16}$$

$$\frac{d[IXa=VIIIa]}{dt} = k_{19}[VIIIa][IXa] - k_{18}[IXa=VIIIa] - k_{21}[IXa=VIIIa][X] + k_{20}[IXa=VIIIa=X] + k_{22}[IXa=VIIIa=X] - k_{25}[IXa=VIIIa] \tag{17}$$

$$\frac{d[IXa=VIIIa=X]}{dt} = k_{21}[IXa=VIIIa][X] - k_{20}[IXa=VIIIa=X] - k_{22}[IXa=VIIIa=X] - k_{25}[IXa=VIIIa=X] \tag{18}$$

$$\frac{d[VIII.lca1]}{dt} = k_{24}[VIIIa] + k_{25}[IXa=VIIIa=X] + k_{25}[IXa=VIIIa] - k_{23}[VIII.lca1][VIII.a2] \tag{19}$$

$$\frac{d[VIII.a2]}{dt} = k_{24}[VIIIa] + k_{25}[IXa=VIIIa=X] + k_{25}[IXa=VIIIa] - k_{23}[VIII.lca1][VIII.a2] \tag{20}$$

$$\frac{d[V]}{dt} = -k_{26}[IIa][V] - k_{44}[mIIa][V] \tag{21}$$

$$\frac{d[Va]}{dt} = k_{26}[IIa][V] - k_{28}[Xa][Va] + k_{27}[Xa=Va] + k_{44}[mIIa][V] \tag{22}$$

$$\frac{d[Xa=Va]}{dt} = k_{28}[Xa][Va] - k_{27}[Xa=Va] - k_{30}[Xa=Va][II] + k_{29}[Xa=Va=II] + k_{31}[Xa=Va=II] \tag{23}$$

$$\frac{d[Xa=Va=II]}{dt} = k_{30}[Xa=Va][II] - k_{29}[Xa=Va=II] - k_{31}[Xa=Va=II] \tag{24}$$

$$\frac{d[mIIa]}{dt} = k_{31}[Xa=Va=II] - k_{32}[mIIa][Xa=Va] - k_{39}[mIIa][AT] \tag{25}$$

$$\frac{d[TFPI]}{dt} = -k_{34}[Xa][TFPI] + k_{33}[Xa=TFPI] - k_{36}[TF=VIIa=Xa][TFPI] + k_{35}[TF=VIIa=Xa=TFPI] \tag{26}$$

$$\frac{d[Xa=TFPI]}{dt} = k_{34}[Xa][TFPI] - k_{33}[Xa=TFPI] - k_{37}[TF=VIIa][Xa=TFPI] \tag{27}$$

$$\frac{d[TF=VIIa=Xa=TFPI]}{dt} = k_{36}[TF=VIIa=Xa][TFPI] - k_{35}[TF=VIIa=Xa=TFPI] + k_{37}[TF=VIIa][Xa=TFPI] \tag{28}$$

$$\frac{d[AT]}{dt} = -k_{38}[Xa][AT] - k_{39}[mIIa][AT] - k_{40}[IXa][AT] - k_{41}[IIa][AT] - k_{42}[TF=VIIa][AT] \tag{29}$$

$$\frac{d[Xa=AT]}{dt} = k_{38}[Xa][AT] \tag{30}$$

$$\frac{d[mIIa=AT]}{dt} = k_{39}[mIIa][AT] \tag{31}$$

$$\frac{d[IXa=AT]}{dt} = k_{40}[IXa][AT] \tag{32}$$

$$\frac{d[IIa=AT]}{dt} = k_{41}[IIa][AT] \tag{33}$$

$$\frac{d[TF=VIIa=AT]}{dt} = k_{42}[TF=VIIa][AT] \tag{34}$$
