# Supplementary material for: Defining the Boundaries of Normal Thrombin Generation: Investigations into Hemostasis
Source: PLoS One. 2012 Feb 2;7(2):e30385. doi: 10.1371/journal.pone.0030385 (PMC3271084; doi:10.1371/journal.pone.0030385)
Supplement: Table S2 — Typical initial coagulation factor concentrations and their normal ranges. (PDF) [file pone.0030385.s005.pdf]

Table S2: Typical initial coagulation factor concentrations and their normal ranges.

Protein Factor	Variable	Initial Concentration (M)	Normal Range* (%)	LETS Population Range# (%)	In house Population‡ (%)
TF	x_1	5 x 10^{-12}	Undefined	Undefined	Undefined
VII	x_2	1 x 10^{-8}	60 – 140	41 –171	76-147
TF=VII	x_3	0			
VIIa	x_4	1 x 10^{-10}	60 – 140		
TF=VIIa	x_5	0			
Xa	x_6	0			
IIa	x_7	0			
X	x_8	1.6 x 10^{-7}	60 – 140	46 –163	83-184
TF=VIIa=X	x_9	0			
TF=VIIa=Xa	x_{10}	0			
IX	x_{11}	9 x 10^{-8}	69 – 151	52 –188	74-151
TF=VIIa=IX	x_{12}	0			
IXa	x_{13}	0			
II	x_{14}	1.4 x 10^{-6}	60 – 140	55 –153	89-152
VIII	x_{15}	7 x 10^{-10}	64 – 232	49 –232	99-193
VIIIa	x_{16}	0			
IXa=VIIIa	x_{17}	0			
IXa=VIIIa=X	x_{18}	0			
VIII.1ca1	x_{19}	0			
VIII.a2	x_{20}	0			
V	x_{21}	2 x 10^{-8}	60 – 140	47 – 302	86-138
Va	x_{22}	0			
Xa=Va	x_{23}	0			
Xa=Va=II	x_{24}	0			
mIIa	x_{25}	0			
TFPI	x_{26}	2.5 x 10^{-9}	46 – 171	46 –170	88-148
Xa=TFPI	x_{27}	0			
TF=VIIa=Xa=TFPI	x_{28}	0			
AT	x_{29}	3.4 x 10^{-6}	88 – 174	63 –125	74-131
Xa=AT	x_{30}	0			
mIIa=AT	x_{31}	0			
IXa=AT	x_{32}	0			
IIa=At	x_{33}	0			
TF=VIIa=AT	x_{34}	0			

*: The normal ranges (Reference Intervals) were determined in 2006/2007 by The Fletcher Allen Health Care Special Coagulation/Hematology Laboratory (FAHC, Burlington, Vermont). They tested equal numbers of normal males and females (n=75 plasmas/gender) with 50 donors supplied from Precision Biologic and 25 donors drawn at FAHC. The reference ranges were calculated using the mean +/- 2SD.
#: van der Meer et al (1997) Thromb Haemost 78: 631-5.
‡: Range of thirty-two apparently healthy individuals recruited from hospital and university staff at Jagiellonian University Medical College, Krakow, Poland.
