# Supplementary material for: Defining the Boundaries of Normal Thrombin Generation: Investigations into Hemostasis
Source: PLoS One. 2012 Feb 2;7(2):e30385. doi: 10.1371/journal.pone.0030385 (PMC3271084; doi:10.1371/journal.pone.0030385)
Supplement: Table S3 — Factor levels for control, hemophilia & warfarin groups (Mean ± SD). (PDF) [file pone.0030385.s006.pdf]

Table S3: Factor levels for control, hemophilia & warfarin groups (Mean ± SD).

Protein Factor	Control* (%)	Hemophilia# (%)	Warfarin$ (%)
VII	108 ± 17	87 ± 29	30 ± 9
X	119 ± 21	110 ± 22	37 ± 10
IX	120 ± 20	107 ± 20	34 ± 7
II	111 ± 15	115 ± 15	29 ± 9
VIII	140 ± 27	0.4 ± 0.4	121 ± 29
V	109 ± 12	105 ± 20	105 ± 14
TFPI	114 ± 17	63 ± 12	111 ± 37
AT	100 ± 14	113 ± 11	104 ± 13

*: Apparently healthy individuals (N=32).

#: Severe hemophilia A individuals (N=16), factor VIII levels ≤ 1% at the time of the blood draw.

$: Warfarin treated individuals (N=65, INR=2.6 ± 0.4).
