# Supplementary material for: Defining the Boundaries of Normal Thrombin Generation: Investigations into Hemostasis
Source: PLoS One. 2012 Feb 2;7(2):e30385. doi: 10.1371/journal.pone.0030385 (PMC3271084; doi:10.1371/journal.pone.0030385)
Supplement: Table S4 — Hypothetical normal range plasma compositions resulting in extreme thrombin generation phenotypes. A rank ordering for each of the 6561 simulations for four metrics is shown with the combination of initial factor concentrations that produced them. (PDF) [file pone.0030385.s007.pdf]

TABLE S4. Hypothetical normal range plasma compositions resulting in extreme thrombin generation phenotypes. A rank ordering for each of the 6561 simulations for four metrics is shown with the combination of initial factor concentrations that produced them.

Time to 2nM IIa (min)	%VII	%X	%IX	%II	%VIII	%V	%TFPI	%AT
2.30	140	140	151	140	232	140	46	88
2.35	140	100	151	140	232	140	46	88
2.38	140	140	151	100	232	140	46	88
2.40	140	140	151	140	232	100	46	88
2.42	140	100	100	140	232	140	46	88
…	…	…	…	…	…	…	…	…
14.03	140	140	69	60	64	60	171	174
14.07	60	60	69	60	64	60	171	174
14.30	100	140	69	60	64	60	171	174
14.52	60	100	69	60	64	60	171	174
14.97	60	140	69	60	64	60	171	174

Peak IIa (nM)	%VII	%X	%IX	%II	%VIII	%V	%TFPI	%AT
792.4	140	60	151	140	232	60	46	88
790.9	140	60	151	140	232	100	46	88
789.8	100	60	151	140	232	60	46	88
789.4	140	60	151	140	232	140	46	88
788.1	100	60	151	140	232	100	46	88
…	…	…	…	…	…	…	…	…
25.0	140	140	69	60	64	60	171	174
24.7	60	140	69	60	64	140	171	174
24.6	100	140	69	60	64	60	171	174
24.4	60	140	69	60	64	100	171	174
23.7	60	140	69	60	64	60	171	174

Max Rate (nM/sec)	%VII	%X	%IX	%II	%VIII	%V	%TFPI	%AT
12.4	140	60	151	140	232	60	46	88
12.3	140	60	151	140	232	100	46	88
12.2	100	60	151	140	232	60	46	88
12.2	140	60	151	140	232	140	46	88
12.1	100	60	151	140	232	100	46	88
…	…	…	…	…	…	…	…	…
0.112	140	140	69	60	64	60	171	174
0.110	100	140	69	60	64	60	171	174
0.107	60	140	69	60	64	140	171	174
0.106	60	140	69	60	64	100	171	174
0.103	60	140	69	60	64	60	171	174

Total IIa (sec•nM)	%VII	%X	%IX	%II	%VIII	%V	%TFPI	%AT
134338	140	60	151	140	232	60	46	88
134256	100	60	151	140	232	60	46	88
134239	140	60	151	140	232	100	46	88
134173	140	60	151	140	232	140	46	88
134146	100	60	151	140	232	140	46	88
…	…	…	…	…	…	…	…	…
8966	60	140	69	60	64	140	171	174
8843	60	140	69	60	64	100	171	174
8734	140	140	69	60	64	60	171	174
8594	100	140	69	60	64	60	171	174
8179	60	140	69	60	64	60	171	174
